# From dissociation to embodied memory through voice: music therapists’ perspectives on vocal music therapy with women survivors of sexualized trauma

**DOI:** 10.3389/fpsyt.2026.1852780

**Published:** 2026-08-04

**Authors:** Aviya Riabzev, Susanne Metzner

**Affiliations:** 1 Levinsky College of Education, Tel Aviv, Israel; 2 Universitat Augsburg Philosophisch-Sozialwissenschaftliche Fakultat, Augsburg, Germany; 3 Susanne Metzner, Leopold Mozart College of Music, Faculty of Philosophy and Social Sciences, University of Augsburg, Augsburg, Germany

**Keywords:** dissociation, embodied memory, sexualized trauma, therapists’ perspectives, trauma-informed care, vocal music therapy

## Abstract

**Introduction:**

Sexualized trauma is frequently accompanied by dissociation, disrupted embodiment, and loss of voice. Although somatic and arts-based approaches are increasingly integrated into trauma care, little is known about how music therapists conceptualize and implement voicework with women survivors of sexualized trauma. Drawing on expert interviews, this study explores therapists’ perspectives on vocal music therapy as an embodied–relational pathway from dissociation toward integration and embodied memory.

**Methodology:**

A qualitative expert interview study employed reflexive thematic analysis. Seven experienced female music therapists (ages ~36–78) from Israel, the United States, the United Kingdom, and Belgium participated in semi-structured interviews (~120 minutes) conducted via video platform and transcribed verbatim. Analysis focused on embodied, relational, and affective dimensions of voicework.

**Analysis:**

Three interrelated themes emerged: (1) *Cutting the Dissociation*—breath, toning, mirroring, and free-associative singing linked fragmented self-parts and mobilized dissociated affect within co-regulated holding fields. (2) *Creating Memories*—titrated vocal engagement fostered bodily reconnection and embodied sensory awareness, supported agency, and enabled the formation of returnable experiential traces; voicework also carried risks of evoking traumatic memories, underscoring the need for careful pacing. (3) *Changing Positions within the Abuse Constellation*—dynamic, vocally enacted movement among victim, perpetrator, bystander, and neglectful caregiver positions illuminated transference–countertransference processes and facilitated shifts from avoidance and symbiosis toward boundaries, including an embodied “no,” and individuation.

**Discussion:**

The discussion integrates three interrelated processes — cutting dissociation, creating embodied memory traces, and transforming positions within the abuse constellation — as a continuous, non-linear movement. Therapists’ vocal presence functioned as a co-regulating holding field supporting clients’ embodied reintegration. Vocal music therapy may ease dissociation, support embodied memory formation, and foster relational repair, while also carrying risks of retraumatization — emphasizing the need for specialized training, ethical sensitivity, and client-centered research.

## 1 Introduction

### 1.1 Trauma, the body, and the need for embodied therapeutic modalities

Sexual violence is a pervasive global phenomenon that leaves profound psychological, emotional, and somatic imprints in assaulted individuals. According to the World Health Organization (1), approximately one in three women worldwide has experienced sexual violence in her lifetime. Such trauma undermines fundamental assumptions of safety, trust, and bodily integrity, fragmenting identity and disrupting the survivor’s sense of continuity (2). This fragmentation often manifests as dissociation, leading to disconnection from bodily sensations, emotional meaning, and one’s own voice, leaving survivors silenced both literally and metaphorically (3–5). Psychological trauma, as Herman (2) proposed, constitutes “an affliction of the powerless,” in which systems of care and integration are overwhelmed, leaving individuals disconnected from themselves and others.

Many survivors meet diagnostic criteria for posttraumatic stress disorder (PTSD) or complex PTSD (CPTSD), the latter reflecting the cumulative impact of prolonged interpersonal trauma (6, 7). The ICD-11 recognizes CPTSD as a distinct diagnosis capturing the sequelae of chronic interpersonal trauma (1, 8). CPTSD encompasses the core PTSD symptoms of re-experiencing, avoidance, and hyperarousal, alongside persistent disturbances in affect regulation, negative self-concept, and disturbances in relational functioning (8–10). These disturbances are frequently expressed through emotional numbing, relational withdrawal, and disconnection from embodied experience, including the voice (11, 12). Verbal psychotherapy, although grounded in language, also relies on preverbal and embodied processes (13, 14). Yet language may not fully reach trauma held somatically or outside symbolic awareness (5, 15–17).

As recognition of these somatic dimensions has grown, body-based and arts-informed modalities have become increasingly integrated into trauma-informed care (18, 19). Music therapy, grounded in relational attunement and embodied sound, offers a nonverbal and affective pathway through which survivors can access, express, and transform implicit emotional material (20–23). Within this framework, sound and vibration bridge somatic, psychological and aesthetic experience (24, 25), laying the foundation for vocal music therapy as a relational and embodied process of repair.

### 1.2 Vocal music therapy: definitions and theoretical foundations

Vocal music therapy involves voicework – i.e. the intentional use of vocal sound through breathing, toning, singing, and improvisation. This is based on a distinctive practice for restoring connections between body, psyche, and relational dimensions (26, 27). The human voice, unlike external instruments, originates within the body itself. It is simultaneously personal and relational, both inner and outer (28–30). Because of this dual nature, voicework is regarded to hold a unique capacity to mediate between fragmentation and wholeness (31, 32).

For survivors of sexual trauma, the voice often carries profound embodied as well as symbolic meanings. Experiences of silencing, voicelessness, and shame have been described as central to the traumatic aftermath (2, 33). In vocal music therapy, two interrelated processes become particularly significant: reclaiming agency using one’s own voice, and giving symbolic form to previously unspeakable material (27, 34, 35). Through guided vocal exploration, clients re-engage bodily presence and relational contact, allowing implicit material to emerge, be held, and gradually transformed within a responsive therapeutic field (31, 32).

The body that was once the locus of violation may also become the instrument for reclaimed agency and expression (36, 37). Vocalizing enables survivors to re-inhabit their bodies, give sound to what was once unspeakable, and experience attuned relational presence (27, 35). As therapist and client attune through tone, rhythm, and breath, co-regulation and mutual responsiveness emerge (22, 38), creating conditions in which dissociative patterns may soften, and new relational possibilities can take shape (39).

In this study, we examine how vocal music therapy integrates the sensory, emotional, aesthetic, and relational dimensions of the voice within a musical framework (27, 40).

As with Vocal Psychotherapy (34, 41), vocal music therapy draws on psychodynamic concepts such as Winnicott’s (42) transitional space and Ferenczi’s (43) emphasis on relational authenticity, alongside neurobiological perspectives on vagal regulation and embodied attunement (39, 44). Through breath, resonance, and prosodic exchange, vocalizing facilitates co-regulation and embodied repair, positioning the voice as a relational medium that bridges physiological, emotional, and interpersonal processes (45–47). Conceptualizing vocal music therapy as a clinical framework positions voicework as an integrated constellation of vocal intervention techniques supporting embodied relational experience, aesthetic perception, and musical expression (40, 48).

### 1.3 Empirical context and knowledge gap

Empirical work suggests that voicework in music therapy supports emotional regulation, expression, and integration (26, 27, 48). However, little is known about how music therapists conceptualize and implement these processes in trauma-focused practice, whether in individual or group contexts. Existing studies describe mechanisms of vocal expression, yet no research has examined how music therapists understand and regulate vocal interventions specifically within their therapeutic relationship with survivors of sexual trauma across cultural or clinical contexts.

Broader outcome research on music therapy for PTSD has grown substantially in recent years, with meta-analyses indicating moderate efficacy relative to inactive controls, though typically comparable to standard psychotherapy and based on evidence of low certainty (49, 50). Group receptive music approaches have also shown promising results specifically among women with abuse-related PTSD and complex PTSD (51). A recent qualitative study explored voicework in music therapy under continuous traumatic situations, finding that practitioners shifted toward regulation-focused, resource-building approaches under ongoing threat (52). However, none of this outcome literature isolates active, voice-based interventions with survivors of sexualized trauma specifically, nor addresses the clinical and relational dynamics of such vocal work.

### 1.4 The present study: vocal music therapy as a medium of relational repair

This study investigates how experienced music therapists understand and implement vocal music therapy with women survivors of sexual trauma. Through in-depth phenomenological expert interviews, it explores how therapists make sense of the embodied, relational, and affective dimensions of voicework across diverse settings. The analysis focuses on how vocal expression links dissociated self-parts, evokes embodied emotional awareness, navigates therapeutic challenges and supports processes of reconnection and integration.

## 2 Methodology

### 2.1 Research approach

This qualitative expert interview study employed reflexive thematic analysis (53, 54), chosen for its capacity to explore shared patterns of meaning across participants while preserving the interpretative role of the researcher and maintaining methodological coherence with the study’s phenomenological and relational orientation. This approach aligns with the study’s aim to explore therapists’ perspectives on the embodied, relational, and affective dimensions of voicework with survivors of sexual trauma. To ensure methodological coherence appropriate to reflexive thematic analysis, reporting was guided by the Reflexive Thematic Analysis Reporting Guidelines (RTARG; 55), grounded in a values-based approach to qualitative reporting (56).

### 2.2 Participants

Seven experienced music therapists (all women, reflecting the demographic composition of clinicians specializing in this field rather than an explicit gender-based inclusion criterion) aged 36–78 (*M* = 50.3), participated in the study. They represented four different countries: Israel, the United States, the United Kingdom, and Belgium, with clinical experience ranging from 12 to 43 years (*M* = 22.6). All participants had formal training in music therapy and specialized training in trauma-informed practice. Each had substantial experience working with women survivors of sexualized trauma in individual and/or group settings.

Participants were recruited through purposive sampling to ensure variation in geographical background and clinical orientation. The departure point was the first author’s professional networks, supplemented by snowball sampling. Inclusion criteria required formal training in music therapy, specialized experience in trauma-informed practice and vocal/voicework, a minimum of 10 years of clinical experience, and substantial direct clinical experience working with women survivors of sexualized trauma; clinicians lacking this profile were not eligible for inclusion. Educational backgrounds varied, though the sample was generally influenced by the pioneering work of either Austin (34) or Newham (57). Participants worked in both individual and group clinical settings.

An overview of participant characteristics is provided in **Table 1** to contextualize the interview excerpts presented below:

**Table 1:** Participant characteristics.

Participant	Pseudonym	Country	Age	Years of clinical experience
P1	Kelli	Belgium	36	14
P2	Fani	Belgium	36	14
P3	Eva	Israel	41	15
P4	Efi	Israel	42	12
P5	Jill	Israel	65	35
P6	Ruth	United Kingdom	54	25
P7	Debora	USA	78	43

### 2.3 Procedure

Data was collected through semi-structured, guideline-based interviews lasting approximately 120 minutes each, conducted via Zoom. The interview guide included open-ended questions exploring participants’ clinical approaches, embodied experiences, relational processes, and ethical considerations when integrating voicework in trauma therapy.

All interviews were audio-recorded and transcribed verbatim. Ethical approval was obtained from Bar-Ilan University’s Ethics Committee, and all participants provided informed consent. Confidentiality, anonymity, and the right to withdraw at any time were guaranteed.

### 2.4 Data analysis

Data were analyzed manually using reflexive thematic analysis (53, 54). The analytic process involved repeated readings of transcripts for immersion, followed by detailed descriptive, linguistic, and conceptual noting by the first author. Codes were generated inductively, allowing patterns of meaning to emerge from the data. These codes were then developed into candidate themes, refined through iterative movement between individual transcripts and the full dataset, and organized into overarching main themes. Interpretative dialogue between the authors supported theme development, refinement, and naming. During the analytic process, selected interview excerpts were identified as anchor citations. These citations were chosen for their representativeness and analytical relevance and were coded according to three levels: main theme, subtheme, and sequential order of appearance within that subtheme (e.g., Q1.2.3 = third quotation in subtheme 1.2), to enable precise cross-referencing across the Analysis and discussion sections. Consistent with the epistemological assumptions of reflexive thematic analysis, sample size was not determined by thematic saturation, a concept more closely aligned with grounded theory’s structural assumptions about data completeness (54).

### 2.5 Reflexivity

The first author, a music therapist specializing in vocal work with survivors of sexual trauma, maintained reflexive memos and supervision notes throughout the research process. Reflexive engagement included regular discussions with the second author, an experienced music therapy researcher specializing in trauma, who served as an external reflective mirror for assumptions, affective responses, and interpretative positioning. This ongoing dialogue supported reflexive awareness and ensured that interpretations remained grounded in participants’ voices rather than researchers’ preconceptions.

### 2.6 Trustworthiness

To enhance methodological coherence and reflexive openness (55, 56)^1^, several strategies were employed. Reflexivity was maintained through systematic journaling and analytic memos, documenting the researchers’ assumptions and emotional responses. An audit trail was kept throughout the analytic process to ensure transparency in interpretative decisions. Although the first author conducted the primary analysis, ongoing reflexive dialogue and peer debriefing with the second author, an experienced qualitative researcher, supported deeper insight and interpretative accountability. Thick description and rich illustrative quotations were used to convey the depth and complexity of participants’ experiences. Both authors remained aware of their positionalities as clinicians and researchers engaged in vocal and trauma-informed work, maintaining reflexive attention to their role in shaping the interpretative process. To further support reflexive openness, the analysis and methodology sections were shared with all participants for member reflection. Five of the seven participants responded, offering their perspectives on the descriptions presented. The remaining two participants did not respond. Both of the non-responding participants cited limited availability due to significant personal and professional life circumstances already during the data collection period. However, the permission for the analysis, interpretation and publication is covered by their signature beneath the informed consent.

### 2.7 Analysis

This analysis focusses on voicework as a technique within vocal music therapy with survivors of sexualized trauma, examining it from therapists’ perspectives without taking other elements (e.g. conversation, instrumental improvisation) of the therapy into account. Voicework was described in general as addressing unprocessed traumatic material and fragmentation of self; by enabling vocal expression, it supports reconstruction and integration. The process is non-linear and organized into three main themes—Cutting the Dissociation, Creating Memories, and Changing Positions within the Abuse Constellation (see **Table 2** for an overview of themes and subthemes).

**Table 2:** Main themes and subthemes.

Main theme	Subtheme
1. Cutting the Dissociation	1.1 Recognizing the unfamiliarity of one’s own voice 1,2 Tolerating uncomfortable sensations through voicework 1.3 Vocal experimenting with incompatible parts of the self
2. Creating Memories	2.1 Touching aversive bodily memories through voicework 2.2 Opening spaces for vocal resonance and replacing negative memories with new experiences
3. Changing Positions within the Abuse Constellation	3.1 Echoes of the perpetrator-victim dynamic in the therapists’ voices 3.2 Shifting vocal positions from avoidance to nurturing

#### 2.7.1 Cutting the dissociation

Most interviewees described voicework as linking disparate self-parts with inner emotions, thereby facilitating integration of dissociated contents.

##### 2.7.1.1 Recognizing the unfamiliarity of one’s own voice

Six of the seven interviewees described voicework as beginning with breath—an active, *moment-to-moment* process that mobilizes “frozen” affect and initiates linkage among self-parts. They explicitly grounded this process in neurophysiological regulation and self-psychological integration, rather than treating it as a detached, purely expressive activity. As Jill explained:

When I breathe in and out, I create movement. [.] In sexualized trauma, emotions are often frozen in time because the assault wasn’t processed and the frontal cortex was detached. Emotions like shock, anger, shame, guilt, and despair get stuck, and using the voice creates movement between different parts of the self. (Jill, Q1.1.1)

Rather than predicting which specific emotions or self-aspects will surface, Jill emphasizes allowing the client’s unique vocal expression—often through singing—to lead. Interviewees also described using clients’ own words as reflective summaries, consolidating new links between inner and outer worlds and among fragmented self-parts.

Voicework connects fragmented, opposing self-parts. When a client sings, many parts emerge and link. As my client Elsa said: ‘I am now part of the world of those who have a voice, and I think I’m building some kind of identity’—what Jung would call the Self. (Jill, Q1.1.2)

A concrete clinical instance of this linkage was offered by Jill, describing a client with a history of complex and sexual trauma within the family:

I can give an example of someone who had experienced complex trauma, including sexual abuse within the family, who drew her disconnection quite literally. She drew her voice like a kind of bladder with a line through it—an empty section, and another part above it. When I showed her and helped her find her chest voice, she felt she was able to make a connection there. She also drew an inner voice, an outer voice, and a blockage. Singing from the chest voice was what made that connection possible. (Jill, Q1.1.3)

Vocal expressions mirror inner parts, and the field where they meet is fluid and vivid—full of shifts and stark contrasts that let fragmented aspects begin to link.

All these parts of the self begin to connect—familiar with unfamiliar, instinctive with playful, even aggressive; like a painting, I see it as a connection between head and body. (Jill, Q1.1.4)

##### 2.7.1.2 Tolerating uncomfortable sensations through voicework

Five of the seven interviewees described a shift from fight–flight–freeze toward befriending emotions, beginning with attention to body signals and steady therapist mirroring. Vocal interventions linked bodily cues with named feelings, clarified inner states, and helped regulate arousal.

Most people are caught between fight–flight–freeze and rarely reach the ‘green’ safe zone, which is why they dissociate. We help them feel what signals their body is giving and then go to the emotions that go with those signals—learning to be friends with their emotions and with themselves. (Kelli, Q1.2.1)

For example, if I see someone doing this in the session [gestures indicating discomfort], I sing: ‘I see something going on—do you feel what you’re doing? What are you feeling now?’ I constantly mirror to connect with inner feelings that were dissociated. Their singing, breathing, and sounds aren’t just grounding—the vibrations bring up deep, stuck feelings so they can be released. (Kelli, Q1.2.2)

Debora likewise emphasized that voicework recognizes and legitimizes feelings, framing the emergence, acceptance, and containment of previously unmirrored affects as developmental repair.

It’s a developmental process. We need mirroring throughout life. As people heal, new feelings or unseen parts emerge. Especially for survivors of sexualized trauma— they never got to be angry and have that mirrored that that was okay. (…) As babies, it was all by singing; it was non-verbal (Debora, Q1.2.3)

##### 2.7.1.3 Vocal experimenting with incompatible parts of the self

Five of the seven interviewees described voicework as repairing missed developmental processes. In vocal duets, therapists applied techniques such as mirroring and melodic containment so that unfamiliar affects become thinkable and linkable. Debora emphasized a vocal intervention she calls “Free Associative Singing.” In practice, she repeats the client’s own words *in melody*—both reflecting and holding them—to clarify, legitimize, and connect previously unconscious feelings.

I use a free-associative technique, singing the client’s exact words. If she says “I’m scared,” I sing it back [singing] ‘I’m scared!’. If it doesn’t feel right, she adjusts— “actually, I was frustrated.” There’s no right or wrong. They begin to feel, become aware, and create connections between unfamiliar emotions. (Debora, Q1.3.1)

Ruth likewise emphasized using deeper vocal sounds to reach previously dissociated inner layers and support integration. The clinical goal is to help clients find a new inner place and link it to the rest of the self.

In vocal therapy we aim to reach dissociated mental contents and integrate them; using the voice makes this possible. When we arrive at a deep, inner voice connected to deeper feelings—previously not conscious—getting to know them creates new connections and movement between this new part and the others. (Ruth, Q1.3.2)

Building on this, Ruth highlighted the importance of finding the voice that says *NO*—a congruent, embodied assertion that many survivors of sexualized trauma could not previously access. This marks a significant developmental milestone that integrates emotional, cognitive, and interpersonal capacities.

Meeting the voice that says NO—really NO— with all the intention and a fuller, deeper sound, matching what I want to say, is essential for survivors of sexualized trauma who previously could not raise their voice to say ‘no’. (Ruth, Q1.3.3)

Returning to the same client introduced above (1.1), Jill further described how vocal work directly addressed the dissociative split she observed between bodily sensation, affect, and verbal expression:

There is a real disconnection there—I see it as a disconnection between the head and expression, and between the parts of the body and emotion. Then, as she slowly connects to her voice coming from here [the chest], you can feel it vocally—something much calmer emerges … Through this, suddenly having a voice here means that something that had been closed opens and connects. (Jill, Q1.3.4)

#### 2.7.2 Creating memories

All seven interviewees reported that voicework facilitates a shift from physical stagnation and detachment to reconnection with bodily sensations among survivors of sexualized trauma. In five accounts, this embodied reconnection enabled clients to re-experience the self and consolidate new, previously inaccessible memories. Therefore voicework inevitably brings clients into contact with aversive bodily memories. The interviewees’ accounts show a careful navigation (2.1) in order to work through difficult material, rather than bypassing it. A cautious reopening of spaces allows accessible memories to take shape (2.2).

##### 2.7.2.1 Touching aversive bodily memories through voicework

All seven interviewees reported that voicework—through breathing, sounding, and toning within a slow, titrated, co-regulated musical frame—helps clients re-engage bodily sensation and supports reconnection, restoring a sense of ownership and mastery and recovering from the effect of sexualized trauma, such as physical stagnation, detachment, and negative body image. Therapists emphasized careful attunement and caution to safeguard this fragile process.

Some clients hold an image of the body as dirty, like dirty laundry, after sexualized trauma, and long to get themselves back. Singing, and using the voice, create movement over time as breath and sound release stagnation and re-connect them to the body—integrating body, emotions, and mind and bridging the split. (Jill, Q2.1.1)

Trauma is stored in the body, so even breathing can be traumatic. Some can’t take a deep breath without crying. We go slowly and use toning—focusing on producing a tone rather than on the breath—so they breathe deeper indirectly, helping them reconnect with their bodies. (Kelli, Q2.1.2)

Therapists emphasized co-regulation and containment (e.g., staying on two chords) to create felt safety for breath and sound work, allowing clients to explore their bodies and the emotions stored within them and to become more connected to themselves, as Debora reports:

The whole aspect of singing is that it brings you into your body. I wanted people to become embodied and safe. Some can’t even breathe. I would say: let’s breathe together. They couldn’t even do that and start crying from what they’re holding—pain, sadness, grief, anger. So I always start with breathing; and if they can’t, we work gently to find safe ways to breathe. (Debora, Q2.1.3)

Debora continues to narrate how turn-taking vocal techniques (call-and-response, mirroring) invite playful exploration of clients’ feelings about their bodies, reflect inner states, and heighten awareness of physical sensations—often unlocking a first body-positive anchor and softening rigidity:

One of my hardest sexualized trauma cases was an anorexic client who liked nothing about her body. We sang playfully and I said: ‘I like my feet’. She said: ‘I don’t!’—until I said: ‘I like my ears,’ and she laughed: ‘I like my ears.’ That small point of liking became an entry to the body; playfulness worked with her rigidity. (Debora, Q2.1.4)

##### 2.7.2.2 Opening spaces for vocal resonance and replacing negative memories with new experiences

According to five of the seven interviewees, voicework helps clients re-enter their bodies and re-experience physical sensations. Efi described how producing the voice from different body regions—separately or in combination—links to body-related imagery, heightens awareness, and re-establishes connection between mental and physical sensations.

I worked with a client who had survived intrafamilial sexual assault, helping her discover her chest voice. As she sang, she gradually began to connect with her voice, feeling its resonance both from above—placing her hand on her chest and describing the sound as the howling of a jackal—and from below, from a grounded, deeper place in her abdomen, which she associated with the image of waterfalls. Through this voicework, as she heard and felt the integration of these two resonances into a single, unified voice, something opened within her—reconnecting her body and mind. (Efi, Q2.2.1)

Like Efi, Eva also explains how voicework helps clients connect with their bodies and gain new experiences in their lives, including a sense of control, having choices, and expressing their feelings and needs.

In the beginning, she was often dissociated—the tension was too high, with frequent hyperventilation and physical pain. The work initially focused on calming the nervous system and finding a sense of calm breathing and being. Over a year of vocalizing and singing together, she has become a different person: more present, making eye contact, recognizing when she is tired, and saying so. The change is striking, reflecting the strength of the voicework. She now shows mastery and agency—no longer numb and silent, but expressing her needs: “I need this to regulate,” or “I need to release this feeling.” (Eva, Q2.2.2)

Jill highlights both the power and the potential risks of using voicework to break dissociation and evoke awareness of traumatic memories. She shows how integrating voice and bodywork can reconnect physical sensations with memories of sexualized trauma, fostering deeper understanding. As Jill reflected, this power becomes most tangible in moments when sound evokes embodied memory.

I would say there is tremendous power in voicework. I recall a case in which a client, after working with deep vocal sounds resonating in her stomach and pelvis, remembered sexual abuse she had not been aware of. The connection between voice and body in that area unlocked the memory. This shows that voicework can access intense experiences, including traumatic sexual content, and therefore calls for great caution. (Jill, Q2.2.3)

#### 2.7.3 Changing positions within the abuse constellation

All interviewees noted that severe trauma fragments experience, leaving it difficult to process coherently. This fragmentation often confines the trauma to sensory or motor sensations without forming a clear narrative, disrupting the person’s sense of self-continuity. They described how aspects of sexualized trauma remain inaccessible due to the dissociative dynamics embedded within the *abuse constellation*. Within this constellation, client and therapist may unconsciously shift between internalized roles—victim, perpetrator, bystander, or neglectful caregiver—reflecting the emotional and relational complexity of these identifications. Such roles can shift over time: the therapist may momentarily embody the victim while the client takes on the role of the abuser, and vice versa. The analysis suggests that these shifting positions are not only relational but also vocal—with the therapist’s voice playing a key role in actively reflecting, containing, and transforming movements within the abuse constellation.

##### 2.7.3.1 Echoes of the perpetrator-victim dynamic in the therapists’ voices

Five of the seven interviewees described how voicework illuminated the therapist’s shifting positions within the abuse constellation. They noted that this constellation may be re-enacted in therapy when the therapist momentarily embodies acoustically the attacker, the neglectful parent, or even the victim, while the client re-occupies positions of the victim. Attending to the therapist’s voice, where subtle shifts, such as a softening tone, may mirror the client’s experience of being unheard, provided a way to recognize and transform these dynamics within the relational field. As Efi explained:

Working with survivors evokes complex countertransference processes and very difficult feelings. Sometimes, in the therapy of sexually traumatized clients, you suddenly find yourself becoming the abuser—you don’t know it at first. Little by little, as the relationship develops, I notice a vocal countertransference: when my throat suddenly closes, I, who usually know my voice, realize that I’m feeling what she felt—being with no voice in the world. It keeps reminding me how essential it is to listen to the voice. (Efi, Q3.1.1)

When describing the changing positions of therapists in the abuse constellation, several interviewees spoke of moments when the therapist assumes the position of a “good parent.” In such moments, the therapist’s voice becomes gentle and nurturing—sometimes through singing lullabies originally written for their own children.

Yesterday, I sang a lullaby I had written for my own children, but with her name, because she’s so alone. She was deeply moved and took it home. I see it as a kind of empathy that helps me to understand her—not a harmful countertransference, but a good one. (Kelli, Q3.1.2)

In contrast, therapists also recognized moments when they became *“the parent who doesn’t see,”* when clients felt unseen or blamed themselves. These situations often emerged when the therapist was unable to meet the client’s needs, unintentionally evoking early experiences of parental neglect or betrayal. In such cases, therapists described a wish to reclaim their own voice by singing about the client’s inner voices—expressing themes such as guilt, criticism, or defense—in order to create a clear and protective boundary within the relationship.

A recurring theme between us is guilt—she often feels it’s her fault, that she’s not good enough, that I’m letting her down, just as her mother once did. When I realized it was not my voice, I wanted to find my own voice in the therapy—and help her find hers. So we began to sing about guilt, the inner critic, and the defenses, to let those voices be heard and remind her she’s not alone. (Eva, Q3.1.3)

Through this vocal engagement, the therapist re-established a sense of boundary and containment for both, transforming the dynamic of guilt into a shared experience of recognition and regulation.

Additionally, the interviewees discussed situations in which the therapist herself becomes the victim within the abuse constellation. In such moments, the therapist may feel unprotected and unsafe, as the established boundaries do not always shield her from the client’s intrusiveness.

One of my clients has difficulty with boundaries. She can feel highly intrusive, almost penetrative at times. At one time she was standing at my door at home on a weekend morning while she was drunk. We had a whole process of finding safety for both of us, finding clarity and limits in the relationship. (Fani, Q3.1.4)

Feeling unsafe, the interviewees described moments when they momentarily lost their voices, an embodied reflection of fear and dysregulation. To restore a sense of safety, they used their own voice and breathing to help the client regulate, which in turn also stabilized them.

When I feel my voice closing, I know that using it immediately creates a safe space. When she crosses boundaries, I realize she might be dissociated or in too much tension—hyperventilating, in physical pain from the abuse. The focus is to calm the nervous system, to find calm breathing and more flow of her breath, releasing the feeling. I see the tension go down when we just take time to breathe and make sounds. (Fani, Q3.1.5)

Overall, the therapists described a dynamic movement of voices and roles—from dissociated and fearful positions toward embodied mutual engagement and co-regulation. The voice, initially shaped by fear, guilt, or over-identification, gradually became a medium of connection and repair. As Jill reflected:

It starts from a primary relationship between a mother and a baby within the regressive vocal work. I check what kind of mother to be for her, and then I feel how difficult it is—to be experienced as intrusive or abandoning. This delicate game slowly develops until we both stand and play with our voices and improvise. From a very primary and protected stage, she opened up until she felt she was communicating more mutually. Through this important vocal envelope, the most natural connections were created. (Jill, Q3.1.6)

##### 2.7.3.2 Shifting vocal positions from avoidance to nurturing

Six of the seven interviewees described how, at the beginning of therapy, clients tend to avoid using their voice. Although they come to vocal therapy with a wish to find their voices, vocalizing often feels threatening, as it echoes the patterns of sexualized trauma that occurred within a relationship of intimacy and exposure.

They come to therapy so that I can help them with their voice, but they say: wait, wait, don’t get near me. It’s my most intimate thing. It almost feels like a reconstruction of the sexual assault—it feels intrusive. (Ruth, Q3.2.1)

Interviewees further described the threat clients feel when their own vocal sounds trigger sensory memories of the abuse.

Because of these aggressive memories that attack them every time— these flashbacks, it is very difficult to breathe and make a sound. The voice is, in a way, an erotic instrument: sighs, moans, sounds that can recall the voice of the abuser. These are voices that cannot emerge elsewhere, and at first, it’s very scary. (Jill, Q3.2.2)

Beyond the clients’ sense of threat and avoidance, interviewees also spoke about their own moments of dissociation and voicelessness when working with survivors. Fear, they said, could take over, silencing them in the face of intense transferences and projective identifications.

I was horrified by working with this population—there is so much transference and projective identification everywhere. Sometimes I felt as if I had no voice—and I am someone who knows my voice. I’ve been in all these positions: the perpetrator, the victim, the bystander, the mother who doesn’t see. There was so much projection and countertransference, and sometimes I dissociated myself. (Fani, Q3.2.3)

Gradually, the voice became a medium of holding and safety. Interviewees described beginning with the simplest vocal gestures*—*tones, humming, or syllables*—*to contain and mirror, attuning themselves to their clients and helping them feel secure enough to begin using their own voices.

She looked into my eyes, and we just started doing la-la-la. We sang in unison, and she felt secure—because I figured we couldn’t really make a mistake if it’s that simple. (Debora, Q3.2.4)

As therapy progressed, the voice assumed a nurturing, almost bodily quality, symbolizing a shift from symbiotic connection toward individuation.

I felt like she was a baby bird—and my sound was food. It was feeding her, and it was such a healing thing for her. At first, she didn’t want to separate—she just kept joining me in unison. To me, this reflected a very early attachment injury, she hadn’t experienced a positive merging. Near the end, she was able to harmonize with me, and I felt she was learning to separate. I think I was right—she eventually moved from the locked unit to a facility where she had more freedom. That symbiotic relationship had been very positive for her. (Debora, Q3.2.5)

## 3 Discussion

This expert interview study explores how music therapists who work clinically with survivors of sexual violence describe and reflect on the use of voicework. Both explicit statements and implicitly conveyed knowledge of seven expert interviewees have been reconstructed through qualitative analysis. The aim is to reveal existing clinical and methodological knowledge, including current therapeutic explanatory approaches, and to contribute to the further methodological development and dissemination of vocal music therapy with this clientele. Some preliminary remarks on the distinctive features of our expert interviews are necessary to make our interpretative steps transparent.

The interviewees in our sample have profound clinical experience, which they mobilize and reflect in accordance with general principles of music therapy with traumatized people like safety, exploration, and relational repair (58–60). Because the first author is herself an experienced clinician in vocal music therapy, participants may have related to her as a “co-expert” (61) rather than as an outsider unfamiliar with the field. This shared professional knowledge may have led some interviewees to offer argumentative or explanatory statements—accounting for why they use particular techniques—rather than describing in granular detail what they actually did, said, or observed in session, as they might have done for a non-expert interviewer. This partial lack of descriptive clarity in the narratives cannot be fully resolved either in the analysis or in the discussion.

In all interviews it becomes particularly apparent that as music therapists the interviewees were aware of their clients’ vulnerability and attune their interventions individually according to their current needs and to contextual conditions. In this way they specify some techniques of voicework and significantly expand methodological aspects. It can also be generally noted that in terms of theoretical knowledge on vocal music therapy they share an emergent understanding of the voice as a relational and embodied medium. From many comments, it can be concluded that in their clinical practice they are familiar with the dual status of voice (62, 63), the concept of “third” in the room, when a triadic field is created by sound respectively voice (64) or bridging vocal sound and language (65, 66).

Typically, statements made by clinicians contain ethical, social, psychotherapeutic and personal beliefs in the efficacy of their interventions, which are needed to be successful in treatment. These beliefs should not be questioned fundamentally by the scholarly scepticism of a researchers. However, the possibility of some circularity between participants’ theoretical familiarity and the analytic themes constructed from their accounts had to be kept in mind. We attempted to mitigate this by prioritizing therapists’ concrete descriptions of clinical moments, bodily and vocal experiences, and client responses over their theoretical interpretations wherever possible; however, fully disentangling observation from theoretically-informed interpretation remains an inherent limitation of interview-based research with clinically sophisticated participants.

The interviewees’ specifications on voicework with survivors of sexual violence have been summarized and synthesized in our theme-subtheme system. Our aim was to retain a certain creative impetus in the analysis, to reflect the characteristics of music therapy processes. The theme-subtheme system contains a great variety of topics. Therefore we decided to emphasize certain aspects. Of particular interest in our discussion is most of all the identification of the vocal interventions used, which address the specific physical, psychological and social processes resulting from sexual violence. Therefore, in addition to our psychodynamic framework our theoretical interpretations are guided by increased attentiveness toward

- the embodied and acoustic nature of human vocal utterance and communication (67),
- the significances, relevances and/or valences of body memories (68)
- the roots of reasoning in the body (69) and
- the logic of signs and symbols (70)

Furthermore, we discuss signs, gaps and/or omissions within the narratives in order to identify specific challenges, risks or limitations of voicework with survivors of sexual trauma. This leads us to offering additional practice interpretations based on theory and/or other empirical findings.

**Figure 1:**
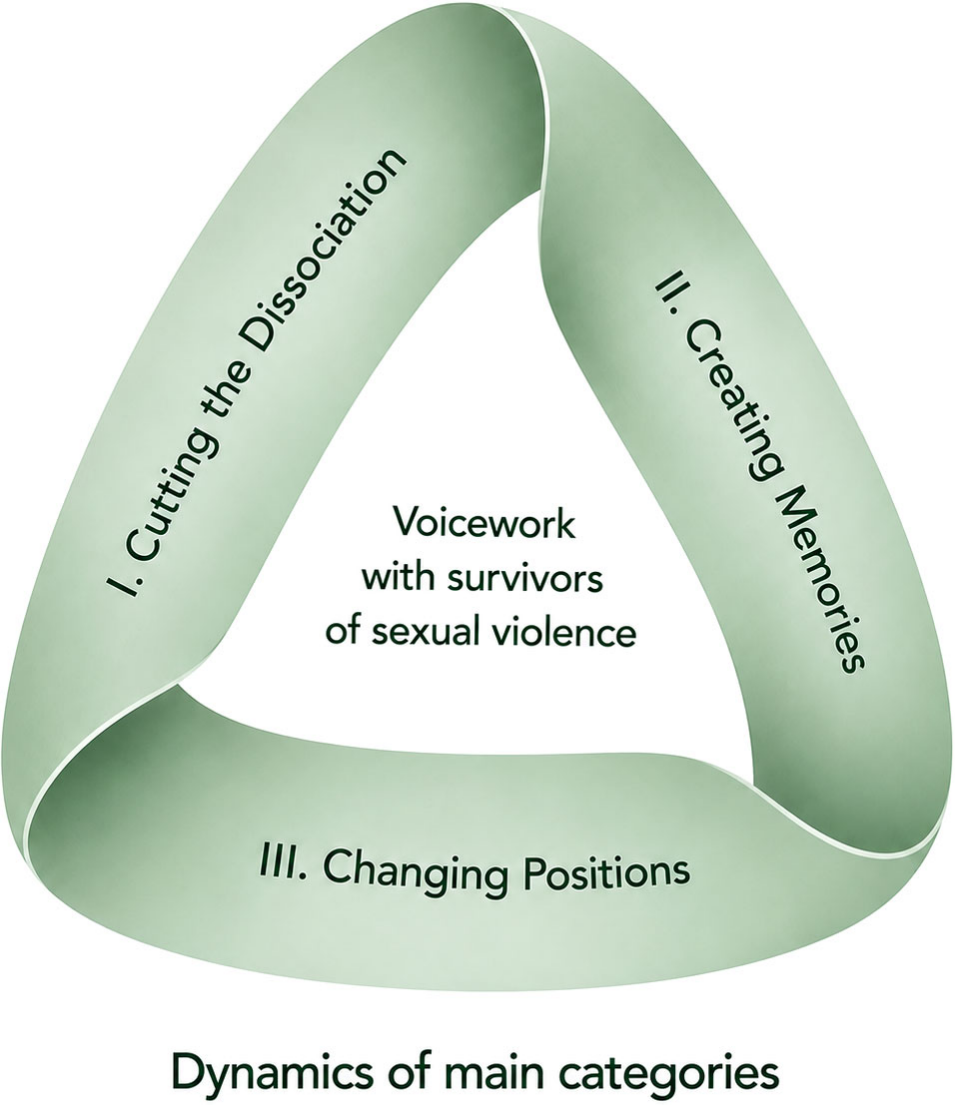
A continuous, one-sided process of voicework, in which the three themes are not sequential stages but aspects of a single ongoing movement.

Our three main themes (Cutting the Dissociation, Creating Memories, and Changing Positions) are interrelated as we illustrated in **Figure 1**. Consequently we are committed to creating transitions between the themes and subthemes in our discussion. Similarly, when referring to anchor quotations, a single quotation might be discussed in relation to more than one theme or subtheme. This reflects the fact that individual quotations often integrate multiple dimensions of experience. Such overlap does not represent an analytical weakness but rather reflects the multidimensional nature of vocal processes in trauma therapy.

### 3.1 From cutting to creating new patterns

The theme “Cutting the dissociation” contains three subthemes that concern different aspects of consciously perceiving and connecting separated self-parts, distorted body images and repressed emotions. The wording of the theme is a paradoxical phrase, since dissociation already cuts experience. We have chosen it to counteract an overly mechanistic conception of dissociation. The psychophysiological process of how dissociation is resolved cannot be directly observed; however, our analysis indicates that dissociated experiences become clinically workable through properties specific to breath and voice. It is noticeable that the interviewees, speaking from clinical and embodied experience rather than diagnostic terminology, do not always differentiate explicitly between repression—in which the original experience was encoded but its affect was subsequently suppressed—and dissociation, in which the experience itself could not be psychically integrated at the time it occurred. Across our data, the material described by interviewees (bodily numbness, fragmentation, “blind spots,” disrupted breath, and self-states inaccessible to verbal recall) is more consistent with dissociative processes than with repression in the narrower psychodynamic sense.

Our analysis suggests that just by breathing consciously in and out, a movement from moment to moment is created, which can mobilize dissociated perceptions caused by sexual trauma (Q1.1.1; Q1.2.2; Q2.1.1; Q2.1.2; Q2.1.3; Q2.2.2; Q3.1.5; Q3.2.2). When the voice comes in, a movement between different parts of the self is created (Q1.1.1) But it can also be the other way round: if conscious breathing is not possible, the path from sounding leads to an indirect intensification of breathing (Q2.1.2).

Interviewees reported that in their clinical practice they help to find the location of the vocal resonance in the body (Q1.1.3), use deeper vocal sounds (Q1.3.3), pick up on singing what is happening in a kind of improvised recitative (Q1.2.2), or use a free-associative technique, singing the client’s exact words (Q1.3.1) to clarify, legitimize, and integrate dissociative states of mind like depersonalization, disturbed body image etc. The concern to transform what was once compulsive into something curious, holdable, and relational meets the explanations published by Austin (34)Fu (71) and Trondalen (64).

The integrative process is promoted or even made possible by the embodied duality of the human voice, which holds an immanent both/and structure of simultaneous inner and outer perception, and of linguistic and pre-linguistic communication. This duality enables contradictory states to coexist audibly rather than collapse into an “either/or” (28, 72). Philosophical perspectives expand this view: the voice is not just sound; rather, it is oriented toward both silence and address, creating an inner–outer chiasm inviting responsiveness (67, 73).

In therapy, such duality provides a pathway beyond dissociation—not by repairing fragments, but rather, by creating a space where opposites can resonate together (64, 73–75). In Winnicott’s terms, a transition is created by the voice, which then serves as a transitional object. In vocal music therapy a potential space is opened, where dissociated parts may be sounded, contained, and related to (42, 76). Jill’s account of a client who drew her disconnection as a literal split between an inner and outer voice, and who experienced these as reconnected once she found her chest voice, offers a concrete illustration of this process (Q1.1.3; Q1.3.4). If this process continues, an affectionate type of relationship to the vocal sounds is created, regardless of whether they are experienced as ugly or beautiful (Q2.2.1). As one client expresses it: “I am now part of the world of those who have a voice, and I think I’m building some kind of identity” (Q1.1.2). In our view the client’s own initiative is crucial here.

Voicework with survivors of sexual violence can create a space without premature interpretation (77, 78). This stance aligns with trauma-informed frameworks that caution against premature sense-making and emphasize titrated, sensorimotor pathways toward integration (78, 79). The sound–language hinge enables a process of graded symbolization (80) allowing the voice to move fluidly from resonance to syllable to word, musical structure or even song (Q1.1.2; Q1.3.1; Q3.1.3). In this way, symbolization unfolds gradually rather than through abrupt leaps. Both presentative (musical) and discursive (linguistic) symbols, in the sense of Langer (70) and Metzner (81), are possible — as are combinations of the two (Q2.1.4).

Beyond symbolization, the voice shapes relational dynamics: it inherently calls for, appeals to, and elicits mimetic response (67). Our findings shows that this responsiveness may be even unavoidable: the therapist cannot remain neutral in the face of the client’s sounding. Vocal-trained music therapists may experience unexpected changes in their own voice (Q3.1.1; Q3.1.3; Q3.2.3). This can be understood as concordant countertransference (82 [1968]), and we tend to associate it causally with the mimetic perception of voice (Q1.3.3). According to Lagaay (67) the voice itself demands reply, moving the therapist, often before conscious choice, into an inner state, or at a position implicitly offered by the client.

This pull carries risks: left unattended, responsivity may slip into repeating trauma-induced dynamics instead of transforming them. In this sense voicework confronts Freud’s (83) repetition compulsion. This has not been reported directly by the interviewees, but their great efforts to minimize the risks of retraumatization are more than evident. Methodologically, this seems anything but simple, because in practice, the dynamic unfolds through micro-interventions: breath entrainment, timbral mirroring or counter-timbre. As one interviewee noted, she constantly mirrors and connects with feelings that were dissociated (Q1.2.2).

In terms of dealing with dissociation, the interviews do not contain many descriptions of more pronounced vocal techniques. Rather, the interviewees tended to place a particular emphasis on the extremely difficult initial phase of therapy, in which it is important to establish the therapeutic relation and build trust despite dissociative states (Q1.2.1; Q2.1.1; Q2.1.2; Q2.2.2). The few explicitly-mentioned techniques seem to be suitable, indeed indispensable, to counteracting emotional numbness and interrupting trauma-related dissociation. Potentially they are capable to sustaining incompatible strands long enough for re-relation to begin.

In a nutshell, cutting dissociation means that instead of re-enacting trauma, the obligation to respond within voicework in real time is meant to enable attuned novelty, disrupting compulsive patterns and opening pathways for symbolization and relation. The inherent duality of voice is both protective and generative. Simply relying on this fact seems more important than working with a large repertoire of vocal techniques. It allows vocal music therapists to work at the edge of the unsayable, making room for dissociated psychic processes to be voiced, heard, and transformed in a gradual process of integration. When worked with reflexively, the mimetic responsiveness becomes a high-resolution source of information about the client’s relational positions, allowing redistribution rather than repetition (84, 85).

### 3.2 From body memory to elementary representation

The theme “Creating Memories” contains two subthemes which describe exploratory and creative processes for regaining body awareness, tolerance toward unpleasant emotions and the sense of the subjective self through voicework. Based on the interviewees’ descriptions, we witness how the quality of memories changes through voicework, i.e. how previously separate or distorted elements can be corrected and creatively integrated. In addition to trauma therapy concepts, there are many possible theoretical references for interpretation, ranging from psychoanalytic theories of memory traces (86), through phenomenological conceptions of the corporeal and intercorporeal unconscious (68), the theory of embodied metaphors (69), and the theory of somatic markers (87), to the now vast body of neurobiological findings on mechanisms of memory and brain plasticity (44, 78). Bringing all of this together while taking into account differences and shifts in perspective would go beyond the scope of this article. We will therefore concentrate on the concept of memory traces with the following basic assumptions:

“Trace” refers to a repeatable vocal imprint, an elementary emergent contour that holds affective memory without yet forming narrative. Traces emerge through subtle vocal forms—pitch arcs, rhythm, and phrasing—that lend shape and temporal contour to otherwise diffuse somatic sensation and/or affect. These bio-psycho-social processes are rooted in very early developmental processes (88–90). Early sound experiences leave embodied imprints that can later resurface in therapeutic voicework, not as explicit memories but rather as affective and acoustic residues. From a phenomenological perspective, traces are never purely individual. Each voice is both personal and intersubjective, formed within cultural soundscapes, resonating with voices of others, and shaped by early, even prenatal, listening experiences and the and cultural anchoring of perception as well as use of the voice (91, 92).

One of our most distinctive findings is that therapeutic voicework makes use of elementary traces aiming to bridge interoception and interpersonally shared resonance, anchoring memory and meaning at the intersection of body, metaphor, and relation (Q1.2.2; Q1.3.3; Q1.3.4; Q2.1.1; Q2.1.2; Q2.1.3; Q2.2.1; Q2.2.3). Within vocal music therapy, sounding itself – whether a hum, sigh, or fragment of melody – can help somatic echoes become tangible (Q2.1.2; Q2.1.3; Q2.2.2). Interviewees report using various vocal techniques to promote this process, such as breathing together (Q2.1.3), making sounds together (Q3.1.5), singing together (Q3.1.3), call and response (Q2.1.4), lullaby singing for the client (Q3.1.2) singing unisono (Q3.2.4).

We already know, from previous studies, that the therapeutic process can build on emergent vocal traces, rather than immediate verbalization, to create a gradual shift from sound toward symbol (34, 57, 93, 94). Embodied vocal traces can be revisited across sessions, allowing memory to be recreated in sound. Associative acoustic imageries such as the “jackal” or “waterfall” (Q2.2.1) show how vocal resonance creates elemental imprints that stabilize affect, reconnecting body and mind. In this way self-regulation improves, and therapeutic interaction is stabilized (Q2.1.4; Q2.2.2) sometimes even in a playful and humorous manner (Q2.1.4; Q3.2.4). From a theoretical perspective, such moments resonate with Stern’s (95) concept of the “present moment” as a critical unit of therapeutic change—brief, affectively charged moments of intersubjective engagement that can become memorable and meaning-bearing without yet forming a coherent narrative.

Working on vocal vibration sensitivity in different regions of the body – chest, stomach, abdomen, pelvis—is described as particularly relevant for survivors of sexual violence (Q1.2.2; Q2.2.1). It is not made explicit, but it is clear from the interviews that these exercises are not forced. They are used with great care within a protected setting to avoid anxiety and keep stress to a minimum, as they can potentially reverse amnesia and bring back memories of the sexual violence suffered (Q2.2.3), entailing the risk of flashbacks, which has been mentioned in the interviews only in other contexts (Q3.2.2).

The interviewees also cited other specific difficulties that need to be reckoned during the therapeutic process with survivors of sexual violence, including: Dealing with dissociative psychic processes (Q2.2.2), “blind spots” (Q1.1.1; Q1.2.1), hyperventilation (Q3.1.5), profound aversion to one’s own body (Q2.1.4), getting in touch with intolerable emotions like shock, anger, shame, guilt, and despair (Q1.1.1), or with the clients’ severe discomfort (Q1.2.1; Q1.2.2) and high tension (Q2.2.2; Q3.1.5). Additionally, interviewees mention that sensations like something stuck in the throat can arise, or that statements like “I have no voice” (Q3.1.1) are enacted vocally or echoed in countertransference (see above). The statement can be understood both literally and metaphorically, which need not be a contradiction. Since it seems to be widespread after sexual trauma, we regard it as an embodied cognitive structure in line with Lakoff and Johnson’s (69) theory of embodied metaphors.

In vocal music therapy obstacles or irritations are not avoided, so that they become audible, tangible acts. Interviewees note that if the therapist picks up on them, clients can literally give form to blockage and then hear or feel it loosen, creating a returnable trace (Q2.1.1; Q2.1.3). Therefore, in addition to the experience of being held and mirrored through micro-practices in voicework that stabilize and extend self-regulation, co-creative working through difficulties seems to contribute significantly to anchor experience in time and meaning and to the formation of a subjective vocal self—a sense of identity shaped through sound (Q1.3.3). In this way voicework moves beyond symptom relief toward broader, long-term goals: If the process continues, clients can start to re-author their life stories and reclaim embodied narrative identity (96, 97).

In a nutshell, memories created in voicework are elemental rather than narrative: they are resonant imprints of affect rather than explicit narrative recollections. Such embodied residues are distinctive in voicework. Interviewees describe how vocalization transforms somatic inscriptions of experience, that manifest themselves in the form of “blind spots,” distorted attitudes and/or avoidances without recognizing, into emerging vocal traces and interactions that can be revisited, varied, worked through and eventually symbolized. In this way creating memories is the groundwork for seizing opportunities offered by life and new ways of relating to self and others.

### 3.3 From victimization to intersubjective flexibility

The theme “Changing positions within the abuse constellation” contains two subthemes, one of which is systemic, the other procedural. Taken together they lead to a process model of triadic relationships. This is not primarily about the voice as a quasi-third that becomes a shared point of reference for the two people in therapy (64), but rather about a psychologically represented multi-person constellation in cases of sexual violence: victim, perpetrator, bystander, or neglectful caregiver. Although it is the concern of both therapist and patient to maintain a relationship free of boundary violations and disrespect and, at most, to refer jointly and consciously to the former experience of violence and to its current consequences, unconscious projections and countertransference in therapy can lead to unwanted identifications with all positions, which is psychologically stressful for those involved (Q3.2.3). As Metzner (81, 98 has already elaborated for improvisational music therapy, the triadic dynamic can be worked through in a beneficial way for the client; the special features of vocal music therapy are therefore of interest here.

Interviewees report emphatically that sudden and initially unnoticed transference-countertransference situations can arise (Q3.1.1; Q3.1.3; Q3.1.4; Q3.1.6; Q3.2.1; Q3.2.3), which provide information about the emotional and interactional events associated with sexual violence and its context. The indicators range from subtle changes in interaction to enactments outside of therapy (Q3.1.4). Therapists have noticed that transference led to the alienation of the interactional situation, manifesting especially in changes in their own voice (Q3.1.3; Q3.1.5; Q3.2.3) or throat (Q3.1.1); this shows how crucial the continuous observation of one’s own physical and sensory processes is for the therapeutic process. From a psychological point of view, the realization of finding oneself in the position of the abuser (Q3.1.1; 23; 24) or in the position of the victim (Q3.1.1; Q3.1.3; Q3.1.4; Q3.2.1) is particularly challenging. When the clients reject voicework, this refusal could maneuver the therapist into the role of the offender (Q3.2.1). Being able to reject, is an important step for survivors of sexual violence. However, when this capacity emerges within the therapeutic relationship itself, it represents an intermediate step that calls for further exploration and working through.

Apart from the emphasis on empathy and building a safe space for co-creativity, the interviewees regard dealing with boundaries as being of vital importance for trauma survivors (Q3.1.4; Q3.1.5). However, the absence of statements from the interviewees in terms of aesthetic qualities suggests that there is no sound, neither vocal nor instrumental, that clearly defines boundaries, an observation consistent with the character of presentative symbols (70, Chapter 4). Even the absence of sound is ambiguous. However, structural means –change of intervention technique (Q1.2.2; Q3.2.4), focusing body signals (Q1.2.1; Q2.1.1), turn take singing/call-and-response cycles (Q2.1.4)–offer an alternative method for boundary setting.

Nevertheless, voicework seems to be beneficial for transitioning to one of the elementary, language-related components of human cognition: negation. Discovering and incorporating the word ‘no’ is an essential experience for those who could not raise their voice before. A clear ‘no’ requires language; credibility, however, is a question of sound and body (as well as context). Our analysis indicates that voicework lends the necessary credibility to the verbal ‘no, especially by working on a deep pitch and achieving a free vibration in the body (Q2.1.1). The ability to negate is a form of boundary setting and abstraction alike and thus provides freedom in several respects. Voicework with survivors of sexual violence, regarded as a method oscillating between sound resonance and words (99, 100), enables experiences with undefined or ambiguous meanings as well as with consolidation and definiteness.

The situation is quite different when the therapist takes on the maternal position. For female therapists, it seems natural to identify with her because the therapeutic task is commonly understood as nurturing (Q3.2.5), supporting (Q1.3.1), soothing (Q3.1.2), mirroring (Q1.2.3), which are mainly associated in society with maternal characteristics. In contrast, the insensitive mother is seen as an evil object–especially in the context of sexual violence: humiliating and blaming (Q3.1.3), intrusive and abandoning (Q3.1.6), ignorant (Q3.2.3). From a feminist perspective, gender stereotypes play a role in these attributions, which are likely to undermine women’s right to their own subjectivity. This perspective remains underexposed in the interviews. However, the interventions indicate that the therapists are indeed concerned with the restitution of subjectivity through voicework. Finding one’s own voice refers not only to voicework with their clients in general, but also their own subjectivity (Q3.1.2; Q3.1.3). This provides the client with a positive example and inspiration for her own development. The shared female identity between therapists and clients may itself constitute a relational and vocal resource within the therapeutic encounter. Several interviewees’ accounts—particularly those describing maternal vocal positions (Q3.1.2) or embodied identification with the client’s experience of voicelessness (Q3.1.1)—suggest that gender-concordant attunement facilitated specific forms of vocal mirroring rooted in shared embodied and developmental experience (e.g., maternal–infant vocal exchange). This raises the question of whether male or non-binary therapists would access comparable relational pathways through different vocal or embodied means, or whether gender concordance constitutes a specific, rather than general, therapeutic mechanism in this population. The transferability of the present findings to therapists of other genders therefore remains an open empirical question. However, as long as the mother position is experienced only in binary terms: either all-good or all-evil, without reflecting on the sociocultural attributions that shape these polarities, it remains a highly ambivalent and under-equipped position (81).

Of particular interest in our discussion is the identification of more vocal interventions, which address the change of positions. What they all have in common is an activity toward what is perceived as unpleasant, unfamiliar or even scary (Q3.2.2) on the therapist’s side instead of avoidance (27).

In that sense, one strategy is not to avoid the maternal position described above as ambivalent, but rather to explore it in “a delicate game” as thoroughly as possible (Q3.1.6). This serves to give the client the opportunity to clarify diffuse feelings and develop behaviors that allow her to distance herself from them. In another case, voicework is referred to as means to learn separation and to leave the symbiosis and/or dissolve dependencies (Q3.2.5).

Vocal expressions that seem erotic are mentioned only once and initially identified with the perpetrator, resulting in a tendency toward negative connotations (Q3.2.2). However, as the interviewee’s statement also insinuates, sighing and moaning can become an exploratory venture that offers the opportunity to separate these vocal sounds from the experience of sexual violence and regain access to one’s own sexual pleasure. Thinking ahead, this is anything but marginal, because in most cases, at an advanced stage of therapy, the issue of one’s own involvement in sexual violence will be addressed, whereby feelings of guilt (to be distinguished from actual guilt) can play a significant role in blocking sexual desire. This can be prepared for at an early stage in a protected environment where vocal expressions outside of everyday conventions are permitted.

The goal of vocal music therapy cannot be permanent liberation, but rather the development of a behavioral repertoire for regulating tension. The experiment with pleasurable sounds seems to be particularly suited to freeing oneself from the stigma of being a victim of violence, whether this happens in a regressive manner (Q3.1.6; Q3.2.4) and/or through experiments with musical togetherness (Q3.1.5), as long as they are contextualized with interventions similar to those outlined previously. In this case the reported results are primarily natural connections (Q3.1.6) or new harmonic ways of vocalizing together (Q3.2.5).

In a nutshell, therapeutic voicework positions the abuse constellation not as a static representation, but rather as a dynamic, audible negotiation. The re-enactment is often first sensed as a vague strangeness that later crystallized into clearer meaning. Actively dealing with the transference-countertransference constellation enables redistribution rather than repetition: positions could be co-sounded and transformed without collapsing into identification or avoidance (27, 34, 64, 84, 85). The restoration of one’s own (vocal) subjectivity is both the method and of voicework and its goal. Importantly, the emerging vocal subjectivity does not belong solely to the individual psyche; rather, it is shaped within relational and socio-cultural matrices.

### 3.4 Limitations and future directions

Despite the study’s contributions, some limitations warrant consideration. First, the small and intentionally homogenous sample—limited to seven highly experienced clinicians—fits the epistemology of Reflexive Thematic Analysis (RTA) but constrains the generalization of identified themes. In addition, all participants identified as women, which may limit the transferability of analysis to therapists of other genders, particularly given the gendered dynamics of voice, embodiment, and sexualized trauma. This question is explored further in the Discussion, where we consider how gender-concordant attunement may constitute a specific therapeutic mechanism in this population.

Second, although the participants represented four national contexts, the analysis did not explicitly address cultural meanings of voice, embodiment, trauma, or relational norms. Given the small sample size, any comparative analysis is not feasible. Future research would certainly benefit from a systematic exploration of the cultural influences of vocal expression.

Third, the study relied on therapists’ retrospective accounts rather than direct observations of clinical encounters. Such self-reports may be shaped by memory, professional identity, or idealized narratives. However, it can be rejected, that the interviewees put into background obscuring moments of rupture, uncertainty, or discomfort that emerge during voicework within trauma therapy as Scrine & Koike (101) insinuated. The interviewees described moments of acute distress and risk (e.g., flashbacks, hyperventilation, the unexpected emergence of previously inaccessible abuse memories). The therapists’ apparent caution and care in these moments was evident throughout the narratives, though the use of systematic safety protocols was not the objective of our inquiry, checking this during the interview would have risked inhibiting frank disclosure. As consequence of our study, future research would benefit from process-oriented designs, including audio- or video-based observation of sessions, to examine therapist’s vocal micro-interventions, moment-to-moment responsivity, and enactments as they unfold in real time.

Fourth, the study accessed only the therapists’ perspectives. Our research cannot replace direct observation of vocal music therapy. Clients’ lived experiences of vocal connection, dissociation, embodied memory, and relational transformation remain largely unknown. Understanding how survivors themselves make sense of vocal processes is essential, particularly given the potential for voicework to evoke deeply embodied or preverbal material. Client-centered qualitative designs could offer crucial insight into how vocal interventions are experienced from within, including moments of safety, risk, or destabilization. Additionally, our analysis of the expert interviews suggests that measurable changes in the areas of body image, body-self-relation, self-esteem and/or sense of coherence can be considered as predictors of a reduction in PTSD symptoms.

Collectively, these limitations underscore the need for multimethod research, including observational studies, client-centered designs, and mixed-method approaches. In addition, interdisciplinary collaboration with neuroscience, developmental psychology, and embodiment research may help clarify how vocal immediacy, prosody, and resonance contribute to affect regulation and memory formation in vocal music therapy with trauma survivors.

## 4 Conclusion

This study offers one of the first systematic examinations of music therapists’ perspectives on voicework with survivors of sexualized trauma across diverse cultural contexts. The analysis reveals how vocal music therapy can ease dissociation, support embodied memory formation, and transform relational positions within the abuse constellation. At the same time, the immediacy of the voice demands careful pacing, ethical sensitivity, and specialized training. By articulating practice-based knowledge that has largely remained implicit, this study positions voicework as a distinct therapeutic pathway within trauma-informed music therapy—one that holds the potential to foster embodied presence, agency, and relational repair.

## Data Availability

Access to the dataset is subject to ethical review and researcher discretion, given the sensitive nature of the clinical material discussed in the interviews. Requests to access the datasets should be directed to AR, aviyak@gmail.com.

## Footnotes

1 Braun and Clarke (2024a, 2024b) distinguish between positivist quality criteria — such as rigor, triangulation, and member checking for accuracy — and Big Q qualitative quality practices grounded in methodological coherence and reflexive openness. The present study aligns with the latter framework.
