# Supplementary material for: From dissociation to embodied memory through voice: music therapists’ perspectives on vocal music therapy with women survivors of sexualized trauma
Source: Front Psychiatry. 2026 Aug 4;17:1852780. doi: 10.3389/fpsyt.2026.1852780 (PMC13482854; doi:10.3389/fpsyt.2026.1852780)
Supplement: Supplementary file 1 [file Supplementaryfile1.docx]

**Supplementary Material 1 — Interview Guide**

**Part I: Context Information and First Associations**

- Can you please tell me some data about your training (where, when?), and your vocal training?
- When you think about vocal music therapy with women who survived sexual abuse — what comes into your mind first?
- When did you start your vocal music therapy work and in which context?

Do you still work in this field? Since when / how many women? / When did you stop, and why?

How do clients come to you? Who pays?

- What is the setting of your vocal music therapy — duration, frequency, equipment of the room?

**Part II: Description of Therapeutic / Vocal Technique**

- Which techniques do you use (apart from vocal music therapy)?
- Which techniques do you use within vocal music therapy?

What are the reasons for using them?

**Part III: Going into Details and Deepening Reflection**

- Can you describe a therapeutic process with one of your clients?
- What did you learn from this?
- Is there a development of your technique?

**Part IV: Generalisation — Professional Meta Level**

- More generally: Which of the client’s main needs were addressed in your clinic, and how did you address those needs?
- Can you please tell me about maybe typical processes / relationships within vocal therapy with women who survived sexual abuse?
- What are your short- and long-term therapeutic goals when treating women who survived sexual abuse?
- What do you consider a successful treatment?
- Why, in your opinion, might vocal therapy help women who were sexually assaulted?

**Part V: Problems, Failure, Contraindication**

- Which techniques did you try and turned out not to be adequate?
- Are there any complications that one has to have in mind when working with vocal therapy?
- Do you think there are factors that indicate contraindication to the use of vocal therapy?

**Part VI: Closing**

- Which advice would you give a colleague who wants to start vocal therapy with this clientele?
- If you think about our interview: what would you like to tell me?

**Supplementary Material 2 — Consent Form**

**Music therapists’ perspective regarding vocal therapy with women coping with sexual abuse, 2022**

**Hello,**

**We, Dr. Aviya Riabzev (interviewer, researcher) and Prof. Dr. Susanne Metzner (scientific supervisor), are conducting this qualitative study based on Expert Interviews. We aim to explore the professional experience of music therapists using vocal improvisational intervention with sexually abused women coping with CPTSD. We thank you for your participation in the study. Your contribution is extremely important for the visibility of professional expertise and the further development of specific music therapy techniques.**

**The research includes participation in one interview. To this end, we would be grateful if you could fill in the following details expressing your consent to participate in the study.**

**I am the undersigned ____________________________ ID no. ____________________**

**I hereby confirm with my signature ____________________________ that:**

**A. I was asked to take part in the interviews, all while maintaining complete anonymity.**

**B. I was not offered any compensation or benefit for my participation in the procedure.**

**C. It was made clear to me that my participation is voluntary and not compulsory, that I will be entitled not to participate or to stop my participation at any stage, and that non-participation or discontinuation will not result in any harm to me.**

**D. After all the above was explained to me, I expressed my consent of my own free will to participate in the interview.**

**Date: ____________________________**

**Name and Surname: ____________________________**

**Signature: ____________________________**

**The undersigned interviewer Dr. Aviya Riabzev verified that the undersigned above understood the details stated in this consent form, that all the information provided will be anonymous, confidential, and accessible only to the researchers, and that the data will be stored on a safe server, secured and encrypted. After verifying all the above, I confirm that the undersigned signed their consent to participate in the interview.**

**Date: ____________________________**

**Signature: ____________________________**

**For any questions, please contact us:**

**Dr. Aviya Riabzev**

**Email: aviyak@gmail.com | Phone: +972-50-698-1515**

**Prof. Dr. Susanne Metzner**

**Universität Augsburg, Leopold-Mozart-Zentrum, Grottenau 1, D-86150 Augsburg**

**Email: susanne.metzner@uni-a.de**

**Supplementary Material 3 — Participant Information Sheet**

*Dr. Aviya Riabzev | Email: aviyak@gmail.com*

*Prof. Dr. Susanne Metzner | Universität Augsburg, Leopold-Mozart-Zentrum, Grottenau 1, D-86150 Augsburg | Email: susanne.metzner@uni-a.de*

**Study title:** Music therapists’ perspective regarding vocal therapy with women coping with sexual abuse.

**Invitation to Participate in the Above Study**

We would like to invite you to take part in our research. However, before you decide, it is important that you understand why the research is being done and what it will involve for you. Please take time to read this information and discuss it with others if you wish. Please ask us if there is anything unclear or if you would like more information.

Your participation in this research is entirely voluntary. If at any point you wish to no longer take part in the study, you have the right to withdraw at any time, and there will be no pressure to stay.

All the information you will give will be anonymous and confidential and only used for the purpose of this study, and will only be accessible to us. The data will be stored only on the interviewer’s computer and on a safe server of Augsburg University and passed between those machines only in encrypted form. No other person but us (Susanne and Aviya) will have access to any of the information you provide.

Please take time to decide whether you wish to take part.

**What is the Purpose of the Study?**

This qualitative study based on Expert Interviews aims to explore the experience of 15 music therapists using vocal intervention with sexually abused women coping with CPTSD. Such research will increase our understanding of the therapeutic techniques and processes, their meaning to clients from the therapists’ perspective, and their potential to promote wellbeing among women coping with sexual abuse.

**What Does the Study Involve?**

The participation in the study involves a semi-structured narrative interview with Aviya Riabzev. The interview guide will include questions regarding the participants’ vocal work with sexually abused women and their perspectives, theoretical approaches, clinical experiences, thoughts, and feelings regarding their work. All personal data given in the interview will be treated as completely confidential.

The interview will be conducted online using the Zoom platform and will last approximately 90 minutes. The interview will be videotaped. All recordings will be destroyed once transcribed and anonymized, or at a maximum of two years after the interview takes place. The data will be analyzed according to the interpretative phenomenological analysis approach.

Unless the participant states otherwise, a copy of the transcript will be sent to them, asking for their consent on whether all parts of the interview may be used, or whether the interviewee would like to erase or change specific passages. If the participant does not wish to read the transcript, they should state this either before the interview or after being offered to receive it. Once the interviewee gives their consent regarding the transcript, they will no longer be able to withdraw, and the researchers are permitted to use the provided data.

**What are the Possible Risks and Benefits of Participation?**

Participating in the research may cause emotional distress while sharing certain parts of the therapy process or situations involving sexually abused women. However, we expect no negative side effects.

The benefits of participation may include the opportunity to share and reflect on certain therapeutic situations in a confidential dialogue at a deeper level, and to gain different perspectives or insights. Moreover, there is a lack of research in the music therapy with trauma survivors’ field, particularly regarding vocal work with sexually abused women survivors, although it has been found effective. Hence, the current study aims to explore the meaning of vocal music therapy from the therapists’ perspective, its potential to promote wellbeing among sexually abused women survivors, and to extend knowledge in the field.

**What is Our Guarantee?**

The participants can withdraw at any time until they consent to use the interview after reading the transcript, and their participation is completely voluntary. Moreover, all the information they provide will be destroyed after the interview is transcribed and anonymized, or at a maximum of two years later.

All the information you will give will be anonymous, confidential, and accessible only to us (Susanne and Aviya). The data will be stored on a safe, secured, and encrypted server.

Thank you very much for your time and help,

Dr. Aviya Riabzev & Prof. Dr. Susanne Metzner
